# Application of Multi-Criteria Decision Analysis (MCDA) to Prioritize Real-World Evidence Studies for Health Technology Management: Outcomes and Lessons Learned by the Canadian Real-World Evidence for Value of Cancer Drugs (CanREValue) Collaboration

**DOI:** 10.3390/curroncol31040141

**Published:** 2024-04-01

**Authors:** Pam Takhar, Marc Geirnaert, Scott Gavura, Jaclyn Beca, Rebecca E. Mercer, Avram Denburg, Caroline Muñoz, Mina Tadrous, Ambica Parmar, Francois Dionne, Darryl Boehm, Carole Chambers, Erica Craig, Maureen Trudeau, Matthew C. Cheung, Joanne Houlihan, Valerie McDonald, Petros Pechlivanoglou, Marianne Taylor, Eric Wasylenko, Wiesława Dominika Wranik, Kelvin K. W. Chan

**Affiliations:** 1 Ontario Health (Cancer Care Ontario), Toronto, ON M5G 2L3, Canada; pam.takhar@ontariohealth.ca (P.T.); scott.gavura@ontariohealth.ca (S.G.); jaclyn.beca@mail.utoronto.ca (J.B.); rebecca.mercer@sunnybrook.ca (R.E.M.); caroline.munoz@ontariohealth.ca (C.M.); 2 CancerCare Manitoba, Winnipeg, MB R3E 0V9, Canada; mgeirnaert@cancercare.mb.ca; 3 Canadian Centre for Applied Research in Cancer Control, Toronto, ON M5G 2L3, Canada; 4 Evaluative Clinical Services, Sunnybrook Research Institute, Toronto, ON M4N 3M3, Canada; matthew.cheung@sunnybrook.ca; 5 Division of Haematology/Oncology, The Hospital for Sick Children, Toronto, ON M5G 1X8, Canada; 6 Leslie Dan Faculty of Pharmacy, University of Toronto, Toronto, ON M5S 3M2, Canada; mina.tadrous@utoronto.ca; 7 Women’s College Hospital, Toronto, ON M5S 1B2, Canada; 8 Division of Medical Oncology & Hematology, Department of Medicine, Sunnybrook Health Sciences Centre, Toronto, ON M4N 3M3, Canada; ambika.parmar@sunnybrook.ca (A.P.); maureen.trudeau@sunnybrook.ca (M.T.); 9 Temerty Faculty of Medicine, University of Toronto, Toronto, ON M5S 1A8, Canada; 10 Prioritize Consulting Ltd., Vancouver, BC V6S 2B3, Canada; fdionne@telus.net; 11 Saskatchewan Cancer Agency, Regina, SK S4W 0G3, Canada; darryl.boehm@saskcancer.ca; 12 Alberta Health Services, Calgary, AB T5J 3E4, Canada; carole.chambers@albertahealthservices.ca; 13 New Brunswick Cancer Network, Fredericton, NB E3B 5G8, Canada; erica.craig@gnb.ca; 14 Nova Scotia Health, Halifax, NS B3S 0H6, Canada; joanne.houlihan@nshealth.ca; 15 Independent Patient Representative, Toronto, ON M6G 2V3, Canada; vmcdonald356@gmail.com; 16 Child Health Evaluative Sciences, The Hospital for Sick Children, Toronto, ON M5G 1X8, Canada; petros.pechlivanoglou@sickkids.ca; 17 BC Cancer, Kelowna, BC V1Y 5L3, Canada; mtaylor@bccancer.bc.ca; 18 Department of Oncology, University of Calgary, Calgary, AB T2N 1N4, Canada; ericwasylenko@platinum.ca; 19 John Dossetor Health Ethics Centre, University of Alberta, Edmonton, AB T6G 2R7, Canada; 20 Department of Public and International Affairs, Faculty of Management, Dalhousie University, Halifax, NS B3H 4R2, Canada; dwl@dal.ca; 21 Department of Community Health and Epidemiology, Faculty of Medicine, Dalhousie University, Halifax, NS B3H 4R2, Canada

**Keywords:** oncology, decision-analysis, health technology assessment, multi-criteria decision analysis

## Abstract

Multi-criteria decision analysis (MCDA) is a value assessment tool designed to help support complex decision-making by incorporating multiple factors and perspectives in a transparent, structured approach. We developed an MCDA rating tool, consisting of seven criteria evaluating the importance and feasibility of conducting potential real-world evidence (RWE) studies aimed at addressing uncertainties stemming from initial cancer drug funding recommendations. In collaboration with the Canadian Agency for Drugs and Technologies in Health’s Provincial Advisory Group, a validation exercise was conducted to further evaluate the application of the rating tool using RWE proposals varying in complexity. Through this exercise, we aimed to gain insight into consensus building and deliberation processes and to identify efficiencies in the application of the rating tool. An experienced facilitator led a multidisciplinary committee, consisting of 11 Canadian experts, through consensus building, deliberation, and prioritization. A total of nine RWE proposals were evaluated and prioritized as low (n = 4), medium (n = 3), or high (n = 2) priority. Through an iterative process, efficiencies and recommendations to improve the rating tool and associated procedures were identified. The refined MCDA rating tool can help decision-makers prioritize important and feasible RWE studies for research and can enable the use of RWE for the life-cycle evaluation of cancer drugs.

## 1. Introduction

As decision-making about funding cancer treatments becomes increasingly challenging, real-world evidence (RWE) is gaining significant momentum and recognition for its capacity to inform, support, and strengthen drug funding reimbursement decision-making in cancer and other disease areas [1]. RWE, generated from data of real-world application, has the potential to address evidence gaps and uncertainties present at the time of initial drug funding recommendations. As such, several regulatory and health technology assessment (HTA) organizations are working to incorporate RWE into the life-cycle evaluation of cancer therapies [1,2,3,4,5]. For instance, in Canada, the Canadian Agency for Drugs and Technologies in Health (CADTH) has launched CoLab, a network of services and partnerships across Canada aimed at supporting post-market drug evaluations through the generation of RWE. As part of CoLab, the Canadian Cancer Real-world Evaluation (CCRE) platform is designed to address uncertainties regarding the effectiveness and safety of funded cancer drugs through RWE [6]. This is especially important for decisions that are not likely to have sufficient classical evidence due to the lack of mature survival data or the inappropriate use of a comparator, for example, to inform them. These analyses will help to understand whether cancer drugs are performing as expected in the real-world and in turn will inform cancer drug funding decisions [7].

The Canadian Real-world Evidence for Value of Cancer Drugs (CanREValue) Collaboration was established to develop a framework for Canadian provinces to generate and use RWE to inform cancer drug funding decisions in a consistent and integrated manner [4]. More specifically, the framework aims to enable the reassessment and refinement of funding decisions that may inform renegotiation, disinvestment, or confirm initial funding decisions by decision-makers across Canada [8,9]. Given the potentially large number of uncertainties that may be addressed through RWE analyses, coupled with the extensive resources (e.g., time, costs, expertise) required to conduct RWE studies, it is imperative to have a mechanism to inform priority setting [10]. According to a recent systematic review, MCDA was most commonly used for priority setting to support decision-making (e.g., coverage, reimbursement, funding) [9]. Multi-criteria decision analysis (MCDA) is a value assessment tool that helps to systematically facilitate transparent and consistent decision-making [10,11,12]. It does this by providing a structured approach in considering key aspects of decisional value while incorporating multiple perspectives. As part of the CanREValue Collaboration, the Planning and Drug Selection Working Group (PDS-WG), one of five WGs established by the collaboration, developed an MCDA rating tool to evaluate uncertainties arising from initial drug funding recommendations and to prioritize potential RWE projects that are relevant and feasible for public payers [8,13].

The CanREValue Collaboration in conjunction with the CADTH Provincial Advisory Group (PAG), an advisory body responsible for providing input on implementation issues to facilitate funding decisions, conducted a validation exercise to evaluate the real-world application of the MCDA rating tool that was developed. More specifically, we sought to apply the MCDA rating tool to a number of RWE proposals varying in complexity to gain insight and to identify efficiencies within the rating tool, the consensus building component, deliberation processes, and prioritization process.

This article provides an overview of the MCDA rating tool, describes the format and procedures involved in the application of MCDA for research prioritization, and reports the outcomes of applying MCDA to RWE proposals. Lastly, several practical recommendations, considerations, and lessons learned for the successful application of MCDA for research prioritization are explored.

## 2. MCDA Validation Exercise: Approach 

The CanREValue Collaboration developed a guideline to inform the validation exercise and ensure that a robust and transparent MCDA process was achieved. The MCDA process includes the application of the MCDA rating tool, including consensus building, deliberation, and prioritizing RWE proposals. The guideline was based on qualitative feedback received from members of CanREValue’s PDS-WG and lessons learned from an initial pilot test. The guideline outlines important factors for consideration, such as the composition of the MCDA committee (e.g., number of participants, roles, and expertise required), format and procedures of the MCDA process (e.g., duration of the meeting, application of the rating tool, deliberation, and consensus building) and methods for data collection and reporting (e.g., documenting consensus scores).

### 2.1. Development of the MCDA Rating Tool

The PDS-WG developed an MCDA rating tool to assess and prioritize potential RWE studies using a stepwise, iterative process previously described by Parmar et al. (2023) [13]. Briefly, the MCDA rating tool consists of seven criteria that define the value of a proposed research topic, each with a unique 3-point rating scale, divided into two groups (Appendix A). Three criteria (Group A) evaluate the importance of an RWE question by assessing the (1) drug’s perceived clinical benefit, (2) magnitude of uncertainty identified, and (3) relevance of the uncertainty to decision-makers. The remaining four criteria, in Group B, evaluate the feasibility of conducting an RWE analysis including (1) identifying appropriate comparator populations, (2) identifying appropriate cases, (3) availability of comprehensive data, and (4) availability of necessary expertise and methodology [13]. Weights were assigned to each criterion using a ranking method, in which PDS-WG members were surveyed to identify the top-rated most and least important criteria [14]. Each criterion began with an equal weight and were adjusted based on the survey responses received. Based on survey results, the most important criteria included relevance of uncertainty, drug’s perceived clinical benefit, and availability of comprehensive data, and the least important criteria included availability of necessary expertise, methodology, and the magnitude of uncertainty [14]. The MCDA rating tool uses an additive approach, in which the unique weight assigned for each criterion is multiplied by the selected rating score and then summed across, generating an aggregated weighted score for an individual RWE proposal ranging from 100 to 300, with a larger score indicating greater value and feasibility [13].

### 2.2. Composition of the MCDA Committee 

Based on the PDS-WG feedback and leveraging the lessons learned from the pilot test, it was determined that the MCDA committee, which is responsible for the application of the MCDA rating tool for the assessment of proposed RWE projects, should include a minimum of ten participants, ideally an odd number, with expertise in drug funding decision-making fulfilling the following roles: clinical expert, payer, methodologist, researcher, patient representative, public member, bioethicist, health economist, PAG member, CADTH pan-Canadian Oncology Drug Review Expert Review Committee (pERC) member, and pan-Canadian Pharmaceutical Alliance (pCPA) member. The CanREValue Collaboration identified individuals across Canada fulfilling these roles as potential MCDA committee members. Individuals were contacted by e-mail explaining the purpose and time commitment of the validation exercise and were invited to participate. A total of eleven pan-Canadian experts were ultimately included as part of the MCDA committee, with some participants fulfilling multiple roles and perspectives. The multi-disciplinary committee of experts included clinicians (n = 3), researchers (n = 2), bioethicist (n = 1), methodologists (n = 1), patient representative (n = 1), health economists (n = 2), pERC members (n = 4), and PAG members (n = 4).

### 2.3. Format and Procedures for the MCDA Process 

#### 2.3.1. Meeting Frequency and Duration

A total of five meetings were planned and held virtually in August 2021, November 2021, February 2022, June 2022, and December 2022 for a duration of 1.5 to 2.5 h each. All meetings were held virtually due to the COVID-19 pandemic and geographical spread of the MCDA committee members.

#### 2.3.2. Selection of Potential RWE Proposals

**Ahead of each meeting, high-priority drug candidates were identified by PAG and were subsequently discussed internally within the CanREValue Collaboration.** Files were identified as high priority based on uncertainties and challenges identified by payers during the HTA and drug funding negotiations. Proposals with varying complexities and disease profiles were selected to ensure that the rating tool was adequate in assessing a range of files important to decision-makers. A list of the RWE proposals and specific RWE questions assessed during the validation exercise is presented in Table 1.

#### 2.3.3. Meeting Materials and Instructions

Meeting materials consisted of an RWE proposal vignette (a document outlining the RWE proposal, research question, and criterion-specific information to support the application of the MCDA rating tool), the CADTH pan-Canadian Oncology Drug Review (pCODR) reimbursement recommendation and evidence package, and the MCDA rating tool. The vignette was populated using information published in pCODR recommendation documents and supplemented as needed with data published from clinical trials and from Ontario administrative datasets. Prior to each meeting, an e-mail was sent to committee members sharing meeting materials and outlining instructions and deadlines. In an effort to ensure that rating tools were completed independently and in the absence of any external influence, members were instructed to independently rate each RWE proposal on its importance (criteria 1–3) and feasibility (criteria 4–7) prior to the MCDA meeting. For each criterion, members assigned a rating on a scale of 1 to 3 and were encouraged to document their reasoning to facilitate discussion and consensus building during the validation exercise meeting. All committee members received the same instructions and were reminded of the importance of their participation during the consensus building, deliberation, and prioritization process. Members were provided an average of two weeks to review and submit their completed rating tool to the project manager. Prior to each meeting, all completed MCDA rating tools were reviewed and meeting agenda and presentation materials were finalized.

#### 2.3.4. MCDA Process: Consensus Building and Deliberation

**Figure 1:**
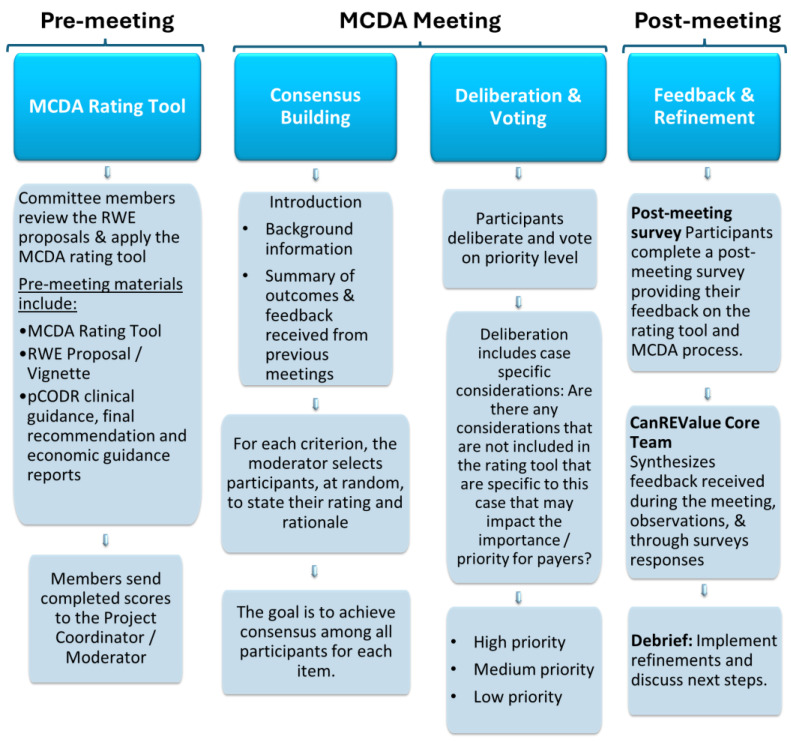
An overview of the MCDA Process.

Each meeting was led and facilitated by the moderator (Chair of PAG, M.G.), with support from the CanREValue Collaboration and the chair of the PDS-WG. Validation exercise meetings were structured as follows: introductions, consensus building, deliberation, prioritization, and closing remarks. Introductions, led by the moderator and chair of PDS-WG, consisted of sharing background information on the development of the MCDA rating tool and providing a summary of the outcomes and feedback received from previous meetings (Figure 1). Following introductions, the moderator led the MCDA committee through consensus building, deliberation, and the prioritization process for each RWE proposal. To begin, the moderator reviewed the information provided for each criterion in the RWE proposal and presented the distribution of scores for each criterion to inform consensus building and deliberation. During consensus building, the moderator selected committee member(s) at random to state their rating and rationale while allowing other members the opportunity to share their perspectives with the goal of achieving a consensus on the rating of each criterion. The moderator elicited dissenting opinions, enabled debate, and sought agreement on the committee’s final score before advancing to the next criterion.

After consensus was achieved for each criterion in the MCDA rating tool, committee members continued deliberations, factoring in case-specific considerations (i.e., additional factors not included in the MCDA rating tool or more nuanced details that were specific to the file and that might impact the importance and/or priority for payers). Using an online voting feature, MCDA committee members voted on the priority level for each RWE question assessed, with the goal of achieving a collective priority level reflective of stakeholder deliberations. Priority levels were defined as high priority (i.e., importance and feasibility are both assessed high), medium priority (i.e., importance or feasibility are assessed high but not both), or low priority (i.e., importance and feasibility are both assessed low). The consensus score for each criterion was recorded using an Excel spreadsheet, which computed a weighted total consensus score for each RWE proposal. In addition, case-specific considerations and the priority level assigned for each RWE proposal were documented.

#### 2.3.5. Feedback and Tool Refinement 

Committee members were encouraged to provide feedback on their experience with the MCDA rating tool, meeting materials, and the overall MCDA process to identify strengths and areas to improve. Feedback was solicited during each meeting and through post-meeting surveys. Following an iterative process, the CanREValue Collaboration, Chair of the PDS WG, and moderator debriefed on the MCDA meeting, providing observations on the experience (e.g., what worked well, and areas that needed to improve) and reviewed committee members’ feedback. Refinements to the rating tool and MCDA process were reflective of these considerations. At subsequent meetings, feedback, compiled into a summary document, was shared along with any adjustments/refinements made to the rating tool and/or the deliberative process. This feedback and refinement process occurred following each meeting. 

## 3. MCDA Validation Exercise: Findings

Eleven pan-Canadian experts forming the MCDA committee participated in a total of five validation exercise meetings. Committee members reviewed and prioritized nine unique RWE questions/uncertainties identified by PAG as presented in Table 2. The application of MCDA proved to be a valuable exercise in assessing the importance and feasibility of potential RWE projects more thoroughly and consistently compared to heuristic evaluations as a majority of the RWE questions were assessed and prioritized as low and medium priority rather than high priority, as initially deemed by PAG.

Throughout the validation exercise, iterative refinements of the MCDA rating tool and deliberative processes focused on increasing the clarity of criterion instructions and rating descriptions. The most significant refinements of the rating tool were made prior to the final meeting in December 2022. At this meeting, committee members re-reviewed the nivolumab RWE proposal, previously assessed in June 2022, in order to explicitly assess the refined rating tool and MCDA process. Overall survival (OS) was the most commonly assessed endpoint during the validation exercise. At the November 2021 meeting, other endpoints were considered, including progression-free survival (PFS), time to next treatment (TTNT) and duration of treatment (DOT). The value of these endpoints and the potential impact on eligibility criteria, renegotiating price, and disinvestment were evaluated. Consistency in MCDA application improved with continued use of the rating tool and with each iteration as criterion instructions and rating descriptions were refined.

Although there were instances of a wide variation in scoring for select criteria, a consensus was consistently achieved for each criterion. Criteria presenting with a wide variation in scoring and generating the most discussion included Criterion 3 (Relevance of uncertainty), Criterion 4 (Identification and assembly of cases and comparator control cohort), Criterion 5 (Sample size for cases and comparator control cohort) and Criterion 6 (Availability of data for covariates and outcomes). Criterion 7 (Availability of expertise and methodology) consistently scored a 3 (of low concern) for each of the RWE proposals assessed. The lack of differentiating rating for this criterion prompted discussion on its value and merit within the tool. 

Through the extensive deliberation and explicit application of the MCDA rating tool, potential RWE projects initially identified as high priority by PAG were most commonly prioritized as low (n = 3; feasibility and importance assessed low) and medium (n = 3; either feasibility or importance assessed high) priority (Table 2). During deliberations for the dabrafenib and trametinib proposal (November 2021), PFS and TTNT were rated lower on importance compared to OS. This rating led to a discussion of unique considerations when evaluating feasibility. Through deliberations, it was acknowledged that these endpoints may not be sufficiently clinically meaningful to change prescribing practices and/or refine eligibility criteria. However, committee members noted the potential for these endpoints to inform renegotiation, provided the outcomes were built directly into the funding agreement. Of the RWE questions evaluated, a single RWE question (duration of treatment) was not prioritized due to time constraints, demonstrating that endpoints are unique and require specific considerations and cannot be assessed simultaneously with other endpoints for the same RWE proposal. Lastly, refinements to the rating tool implemented in the final meeting provided additional clarity and improved the ease of use in the application of MCDA. The refinements did not result in substantial differences in the prioritization of the nivolumab proposal, as the proposal was prioritized as low priority in both the June and December 2022 meetings (Table 2).

Through direct feedback from committee members and observations by the CanREValue Collaboration, several efficiencies and recommendations were identified. Below we summarize the key feedback and recommendations to improve the MCDA rating tool and provide insight into developing an efficient, transparent, and structured MCDA process for the prioritization of RWE projects.

## 4. MCDA Rating Tool: Observations, Feedback, and Lessons Learned

As committee members became more familiar with the nuances of the MCDA rating tool and with continued application, members consistently reported that the tool was easy to use. There was a general consensus that items included in the MCDA rating tool were complete, relevant, and appropriate, as recommended by the ISPOR MCDA best practices, in assessing the importance and feasibility of an RWE proposal [12,14]. As such, no additional criteria were suggested for inclusion. 

During consensus building, inconsistent interpretation and application of the rating tool were observed, resulting in several refinements to the rating tool. The majority of the refinements consisted of improving the clarity of criterion instructions, sharpening the language used to describe the rating descriptions, and explicitly stating performance measures for each criterion to ensure consistent and reliable interpretation and application of the rating tool. The refined MCDA rating tool, reflective of the feedback received throughout the validation exercise, is presented in Table 3. 

### 4.1. Alignment between Criterion Name and Description

The names of certain criteria were considered vague and misaligned with the criterion instructions and rating descriptions, leading to misinterpretation. In response, the name of Criteria 2, 4, 5, and 6 have been revised to ensure both the name and criterion description are aligned. For example, the name of Criterion 5 was revised from “Cases” to “Sample size for Cases and Comparator cohort”. Further, the name of Criterion 2 has been revised from “Magnitude of uncertainty” to “Magnitude of perceived uncertainty,” and the description clearly states common attributes to be considered when ascertaining uncertainty (e.g., immature trial data, use of surrogate endpoints, single arm studies, etc.). 

### 4.2. Improving Clarity on Criterion Instructions and Rating Descriptions

Additional clarification for select criteria was suggested to improve consistency in interpretation and to avoid inadvertent overlap between criteria. Criterion 2, Assessing the magnitude of perceived uncertainty, was initially proposed quantitatively (e.g., <10% variation in either of the following: (a) the confidence intervals around the survival estimates or (b) the upper and lower range of incremental cost effectiveness rations (ICERs) from the pCODR assessment). This approach was found to be difficult to operationalize, since there could be multiple relevant confidence intervals or ICERs presented. In the refined rating tool, a qualitative assessment of whether the primary clinical trial data available are characterized by features of minimal, moderate, or substantial uncertainty is provided with guidance on key elements to consider in decision-making. Through deliberations, it was revealed that certain factors, which were intended to be considered for Criterion 5 (Sample sizes for cases and comparator control cohort) and Criterion 6 (Availability of data for covariates and outcomes), were inadvertently considered when selecting a rating for Criterion 4 (Identification and assembly of cases and comparator control cohort), resulting in an overlap in interpretation between criteria. In the refined rating tool, the instructions for Criterion 4 have been amended, and guidance in selecting an appropriate rating is explicitly stated (e.g., type of cancer, type of treatment received, and clinical characteristics).

Further guidance was also warranted for the evaluation of endpoints other than overall survival (e.g., duration of treatment and time to next treatment) and in assessing non-comparative information. For example, members found it challenging to determine which parameters to use in assessing the perceived clinical benefit (e.g., variation in hazard ratio confidence intervals or variation in median overall survival confidence intervals) and requested additional guidance on the use of upper and lower ranges of ICERs in determining the magnitude of perceived uncertainty (Criterion 2). In response, the instructions and rating descriptions for Criterion 1 (Drug’s perceived clinical benefit), for example, were revised to explicitly describe how committee members are to assess a drug’s perceived clinical benefit (‘based on ratings derived from the European Society of Medical Oncology (ESMO) Magnitude of Clinical Benefit Scale (MCBS)’). Committee members were asked to verify the ESMO-MCBS-endorsed ratings with the information provided in the vignette using the corresponding ESMO-MCBS evaluations forms provided.

### 4.3. Inconsistent Interpretation

During consensus building, it was observed that the interpretation of select criteria (e.g., Criteria 3 and 4) varied based on individual perspectives and assumptions. Some members mentioned that their approach was a literal application of the MCDA rating tool wording, whereas other members were less strict in their interpretation of the description in alignment with their perceived intent of the criterion. We refined ambiguous language to ensure consistent interpretation among diverse stakeholders. For example, the instructions and rating descriptions for Criterion 3 (Relevance of uncertainty) were revised to explicitly state that the criterion is to be assessed with the lens that a health technology life-cycle reassessment mechanism exists to incorporate the findings of a future RWE study into policy decision-making. Refinements to Criterion 3 (Relevance of uncertainty) in the revised version of the MCDA rating tool resulted in a change of rating from low relevance (rating 1) to moderate relevance (rating 2) when evaluating the nivolumab RWE proposal during the June and December 2022 meetings, respectively.

*Initial instructions for Criterion 3: “The objective of this criterion is to assess the relevance of resolving the uncertainty to decision-makers (i.e., what is the likelihood that resolving uncertainty with the findings from the proposed RWE study will alter the funding status or clinical treatment recommendations).”*

*Revised instructions for Criterion 3: “The objective of this criterion is to assess the relevance of resolving the uncertainty to decision-makers. Specifically, users are asked to provide a qualitative assessment on whether the data generated from the proposed RWE study could lead to future policy change (i.e., price re-negotiation, change in funding criteria or disinvestment) assuming that a life-cycle reassessment platform existed.”*

### 4.4. Increasing Transparency on Performance Measures

Through the observation of the validation exercise meetings, it was noted that additional and explicit guidance on the performance measures (quantitative or qualitative metrics referenced in rating descriptions) was needed to ensure consistent application of the rating tool. In the initial version of the MCDA rating tool (Appendix A), performance measures were implied in the descriptions. For example, instructions for Criterion 7 (Availability of expertise and methodology) state ”The objective of this criterion is to evaluate the availability of required expertise and methodologists to conduct the study”, implying that a qualitative assessment by expert opinion is required to assess whether any challenges exist, to what extent, and the potential impact on the feasibility of the proposed RWE question. To improve transparency and consistency in the application of the MCDA rating tool, the type of assessment required (qualitative versus quantitative) is explicitly stated in the name in parenthesis (e.g., Criterion 2 Magnitude of perceived uncertainty (qualitative assessment)) and in the criterion instructions (e.g., users are asked to provide qualitative assessment). 

### 4.5. Value of Criterion 7 (Availability of Expertise and Methodology)

Criterion 7 (Availability of expertise and methodology) was consistently scored as low concern (rating 3) across all nine unique RWE proposals assessed, indicating low concern in the availability of expertise and methodology required to conduct the RWE study. This finding prompted discussion regarding the merit of this criterion within the rating tool, as some committee members felt that the score might artificially skew the aggregated consensus score and that it fails to differentiate potential projects. After careful consideration, it was recognized that the rating tool warrants further application to more complex and methodologically challenging RWE questions (e.g., questions involving tumor agnostic indications may require methods to test for heterogeneity, questions about sequencing may require more complex methods); in which case, this criterion may be less likely to be scored as having low concern.

## 5. MCDA Process (Consensus Building and Deliberation): Observations, Feedback, and Lessons Learned

The validation exercise experience underscored the importance of training stakeholders in the application of MCDA and identified several efficiencies and recommendations. A common misconception of MCDA in relation to decision-making is that the final weighted consensus score determines the decision. This confusion was observed during the initial meeting(s) and was resolved by reinforcing the importance of the deliberative component of the MCDA process and not solely relying on a number. The consensus building and deliberative component allowed for the deeper exploration of the values set out in the rating tool, prompted discussion to incorporate various viewpoints and leverage clinical/payer/methodological insights held by various participants, and created opportunities to raise case-specific considerations unique to the uncertainty/RWE proposal identified [15,16]. As a result of our validation exercise, the majority of the RWE proposals initially viewed as high priority were deemed to be of low and medium priority following a thorough examination and assessment of pertinent values and critical components among a group of diverse stakeholders. 

Although committee members successfully reviewed and prioritized the RWE proposal during our initial 1.5 h meeting in August 2021, additional time for discussion was recommended in the feedback. As such, subsequent meeting durations were increased to 2.5 h. In addition, the initial prioritization categories were outlined as high priority, low priority, and no RWE plan; however, it was identified in the first validation exercise meeting that these prioritization categories were not reflective of stakeholder preferences and could result in forcing an inappropriate priority level, as no moderate priority rating option was provided. After careful consideration, the categories were updated to high, medium, and low priority, respectively.

**Table 3:** Refined MCDA rating tool.

Criteria		Rating Scale			Weight
		1	2	3	
**Group A—Criteria to Assess the Importance of the RWE Question**					
**Criterion 1: Drug’s perceived clinical benefit (quantitative assessment)** The objective of this criterion is to evaluate the perceived clinical benefit of the therapy compared to existing options. The “perceived” clinical benefit is based on the currently available objective evidence, with preference given to primary clinical trial data or indirect comparisons, ^a^ utilized in the setting of single-arm studies or to assess effectiveness in comparison to contemporary Canadian standard-of-care treatments. Assessment of the perceived clinical benefit is based on ratings derived from the European Society of Medical Oncology (ESMO) Magnitude of Clinical Benefit Scale (MCBS) version 1.1 [17]. **Users are asked to provide a quantitative assessment** using the ESMO-MCBS-endorsed rating. The ESMO-MCBS-endorsed rating will be provided in the corresponding vignette (if available); if not, CanREValue will provide a suggested score based on the ESMO-MCBS evaluation forms. Recognizing inter-rater variability, users are asked to *double check* the suggested score using the information provided in the vignette and corresponding evaluations found below. ESMO-MCBS (v1.1) scores: Curative setting: New approaches to adjuvant therapies or potential curative therapies are graded on a scale from A to C, with Grade A indicating a substantial magnitude of clinical benefit. Non-curative setting: Therapies that are not likely to be curative are graded on a scale from 1 to 5, with a Grade 4 and 5 indicating a substantial magnitude of benefit. **Caveats:** Please note that ESMO specifies the use of the lower limit of the 95% confidence interval of the hazard ratio (HR) (instead of the point estimate of HR) when determining the ESMO-MCBS score. Currently, ESMO-MCBS has not been validated for the evaluation of pediatric and hematological malignancies.		Rated using the European Society of Medical Oncology (ESMO) Magnitude of Clinical Benefit Scale (MCBS) v1.0. [17]			17.65
		**Minimal to low clinically meaningful incremental benefit,** as evidenced by ESMO-MCBS v1.1 score of: **(a) Curative setting:** Grade C for new approaches to adjuvant therapy or potentially new curative therapies (please refer to Evaluation Form 1 below for details on rating); or **(b) Non-curative setting:** Grade 1 or 2 for therapies that are not likely to be curative (please refer to relevant Evaluation Form 2a, 2b, 2/c, or 3 below, depending on primary endpoint available for details on rating).	**Moderate clinically meaningful incremental benefit,** as evidenced by ESMO-MCBS v1.1 score of: **(a) Curative setting:** Grade B for new approaches to adjuvant therapy or potentially new curative therapies (please refer to Evaluation Form 1 below for details on rating); or **(b) Non-curative setting:** Grade 3 for therapies that are not likely to be curative (please refer to relevant Evaluation Form 2a, 2b, 2c, or 3 below, depending on primary endpoint available for details on rating).	**Substantial clinically meaningful incremental benefit,** as evidenced by ESMO-MCBS v1.1 score of: **(a) Curative setting:** Grade A for new approaches to adjuvant therapy or potentially new curative therapies (please refer to Evaluation Form 1 below for details on rating); or **(b) Non-curative setting:** Grade 4 or 5 for therapies that are not likely to b e curative (please refer to relevant Evaluation Form 2a, 2b, 2c, or 3 below, depending on primary endpoint available for details on rating).	
**Criterion 2: Magnitude of perceived uncertainty (qualitative assessment)** The objective of this criterion is to assess the degree of uncertainty regarding the endpoint in question. **Users are asked to provide a qualitative assessment** of whether the primary clinical trial data available is characterized by features of minimal, moderate, or substantial uncertainty. There may be features related to the design of the study that may make the results of the study or the expected clinical benefit of the drug to be more uncertain. Common attributes to consider when ascertaining uncertainty include, but are not limited to:		**Minimal uncertainty**	**Moderate uncertainty**	**Substantial uncertainty**	10.6
☐ Immature trial data; ☐ Single arm studies; ☐ Use of surrogate endpoints;	Phase of the trial: Consider whether the trial was early or late phase ☐ Phase I ☐ Phase II ☐ Phase III				
☐ Trials lacking a relevant Canadian standard-of-care comparator; ☐ Existing controversary in the literature or clinical practice;	☐ RWE Applicability: Consider whether there are concerns with generalizability of the trial to the general unselected population in the real world ☐ Other uncertainties Consider other possible sources of uncertainty				
**Criterion 3: Relevance of uncertainty (qualitative assessment)** The objective of this criterion is to assess the relevance of resolving the uncertainty to decision-makers. Specifically, users are asked to provide a qualitative assessment on whether the data generated from the proposed RWE study could lead to future policy change (i.e., price re-negotiation, change in funding criteria, or disinvestment), assuming that a health technology life-cycle reassessment platform existed.		**Low relevance:** As assessed by expert opinions, there is an expected low likelihood for the findings of the proposed RWE study to facilitate a change in policy (i.e., price re-negotiation, change in funding criteria, disinvestment).	**Moderate relevance:** As assessed by expert opinions, there is uncertainty in the likelihood for the findings of the proposed RWE study to facilitate a change in policy (i.e., price re-negotiation, change in funding criteria, disinvestment).	**Substantial relevance:** As assessed by expert opinions, there is an expected high likelihood for the findings of the proposed RWE study to facilitate a change in policy (i.e., price re-negotiation, change in funding criteria, disinvestment).	18.8
**Group B—Criteria to Assess the Feasibility of the RWE Project**					
**Criterion 4: Identification and assembly of cases and comparator control cohort (qualitative assessment)** The objective of this criterion is to assess the likelihood that cases and a relevant historical Canadian comparator cohort can be identified and assembled in at least one Canadian province. A ”relevant” comparator is a group of patients that has been treated according to current Canadian standard of care regimen. To rate this criterion, **users are asked to provide a qualitative assessment** by assessing whether cases and the comparator cohort can be identified based on the availability and completeness of data pertaining to (a) type of cancer (i.e., primary cancer), (b) type of treatment received, and (c) clinical characteristics (i.e., biomarkers, stage, etc.). **Note:** Sample size of the cases and comparator control cohort should not be considered for the rating of this criterion, as it will be considered in the rating of Criterion 5 (Sample size for cases and comparator control cohort). Further, the availability of covariates and outcomes should not be considered for the rating of this criterion, as it will be considered in the rating of Criterion 6 (Availability of Data for Covariates and Outcomes).		**Substantial concern**	**Moderate concern**	**Low concern**	11.8
**Criterion 5: Sample sizes for cases and comparator control cohort (qualitative assessment)** The objective of this criterion is to assess the likelihood that there will be a sufficient sample size of patients receiving the treatment in question (cases) and that a relevant historical Canadian comparator cohort (control) can be identified within a reasonable time frame (i.e., within time to be relevant to the funding decision). **Users are asked to provide a qualitative assessment** by referring to the formal sample size calculation provided in the respective vignette to perform rating for this criterion.		**Substantial concern:** Unlikely to establish a sufficient sample size within a reasonable timeframe to effect meaningful policy change.	**Moderate concern:** Likely to establish a sufficient sample size (as noted in rating scale 3) but additional concern noted (e.g., concern for loss of sample size for cases due to emerging alternative novel therapies with upcoming availability through access programs or public funding).	**Low concern:** Very likely to establish a sufficient sample size within a reasonable timeframe to effect meaningful policy change.	14.1
**Criterion 6: Availability of data for covariates and outcomes (qualitative assessment)** The objective of this criterion is to assess the availability and completeness of data in at least one Canadian province for key covariates and outcome of interest. To rate this criterion, **users are asked to provide a qualitative assessment** by assessing whether key criteria and outcomes can be identified within currently available administrative databases pertaining to data for: (a) Covariates: Relevant patient and/or disease characteristics required to be adjusted and/or accounted for given the non-randomized nature of RWE study design (e.g., consider baseline characteristics listed in Table 1 of the pivotal clinical trial). (b) Outcome of interest *Please note: If the uncertainty is related to cost, users are also to consider data for relevant costing inclusive of total healthcare costs accrued during treatment (e.g., systemic treatment, planned and unplanned healthcare resource utilization).* **Note:** Sample size of the exposed cohort should not be considered for the rating of this criterion (as sample size is considered in the rating of Criterion 5 “Sample size for cases and comparator control cohort”).		**Substantial concern**	**Moderate concern**	**Low concern**	17.65
**Criterion 7: Availability of Expertise and Methodology (qualitative assessment)** The objective of this criterion is to evaluate the availability of required expertise (e.g., clinical experts, data analysts, and methodologists) and methodology to conduct the study. **Users are asked to provide a qualitative assessment.**		**Substantial concern:** Expected challenges to find the necessary expertise and need to develop new methods to conduct the study, with above limitations in data taken into consideration (if applicable).	**Moderate concern:** Expected challenges to find the necessary expertise or need to develop new methods to conduct the study, with above limitations in data taken into consideration (if applicable).	**Low concern:** No expected issues with the availability of the necessary expertise and no new methods required to conduct the study.	9.4

^a^ Please refer to pCODR clinical guidance report for data on previously conducted, relevant indirect comparisons.

The committee felt that consensus building and the deliberation component of the MCDA process was facilitated by a skilled and knowledgeable moderator (M.G.). Committee members throughout the validation exercise acknowledged the expertise of the moderator in helping to evoke thoughtful discussion and support the achievement of consensus among members. To ensure that a single perspective/role did not control the discussion for any criteria, the moderator actively selected committee members at random to share their criterion ratings and considerations while encouraging other members to provide input. This ensured a comprehensive discussion considering multiple perspectives informed the consensus for each criterion and, ultimately, the prioritization decision [16]. It is important to note that consensus was not always easily achieved. In instances where perspectives were quite different from one another, selecting the middle score was the only feasible option in achieving consensus. Although committee members generally expressed comfort with this approach, the implications were further explored when participants were felt to be less confident in the consensus decision (e.g., the consensus score was computed with an alternative choice to assess the impact on the results). We recognize the inherent difference in achieving consensus through a uniform approach versus a consensus achieved by selecting a middle score representing widely divergent views. This challenge further highlights the importance of not relying on a single numerical score in the decision-making process. The deliberative component of our MCDA process encouraged members to factor additional considerations in achieving the final prioritization decision. 

A common barrier identified in the literature in the application of MCDA is the absence of required evidence/information for stakeholders to accurately evaluate criteria and participate in meaningful discussion [16,18]. Information to assess each criterion in the rating tool was derived from credible sources such as CADTH’s clinical and pharmacoeconomic evidence-based recommendation documents. Committee members consistently reported that the meeting materials were helpful in allowing them to score and deliberate in a meaningful way. Further, members found it helpful to have all relevant information summarized for each criterion in a single, succinct document with the option to refer to the original source if additional information was needed for clarification [16]. The time allocated (two weeks on average) permitted participants sufficient time to apply the rating tool while balancing existing workloads and allowed for the retention of critical information, as evident by active participation during meeting discussions. 

The size and composition of the committee were manageable and reflected multiple key perspectives, allowing for rich and meaningful discussion, especially for criteria with wide variation in scoring and interpretation [16]. Members appreciated the transparency in our approach to consensus building and found it helpful to view the rating scores for each criterion presented on screen. Although we strove for full attendance from all eleven committee members during each meeting, full participation was not always achieved due to competing demands and unforeseen circumstances. Our MCDA process was designed to mitigate this impact, as our committee consisted of members representing multiple perspectives and roles (e.g., a member provided perspectives as a clinician and as a member of PAG), and we required members to submit their completed MCDA rating tool prior to each meeting. This approach ensured that broad representation was available in the absence of full participation, and the prior collection of ratings permitted participation from members in their absence as the moderator was able to share their rating selection and rationale with those in attendance to help guide/inform discussion.

## 6. Summary and Future Directions 

Ad hoc prioritization methods fail to accurately identify uncertainties that may be appropriately resolved through the generation of RWE, as evidenced by the methods employed by PAG in initially identifying high-priority files for evaluation. Our proposed MCDA process demonstrated the usefulness of a more thorough, transparent, and structured approach for evaluating key criteria that impact the importance and feasibility of potential RWE studies [16]. Further, our process provoked a rich deliberative discussion of critical components reflective of the values of stakeholders involved in cancer drug funding across Canada.

Interest in using MCDA to support complex cancer drug funding decisions is evolving and generating great interest in Canada and internationally. MCDA is being considered and, in many cases, implemented within international HTA agencies as a mechanism to support transparent, robust, and equitable decision-making as well as in various other decision contexts (e.g., priority setting, clinical decision-making, etc.) [10,15,19,20,21,22,23]. In Canada, value assessment frameworks have been developed to assess the value of oncology therapeutics being funded through public drug programs [24,25]. Although these tools and our proposed rating tool appear similar at the outset (e.g., Canadian context, number of criteria included, criteria titles), there are considerable differences to note. First, the underlying decision context is not comparable between the existing tools and our proposed MCDA rating tool [12]. We have developed an MCDA rating tool specifically to assess the importance and feasibility of conducting RWE studies to support the reassessment and refinement of cancer drug funding decisions in the Canadian context. Second, although there are similarities in the title of select criteria, such as clinical benefit and feasibility, the rationale and description of the criteria included within our rating tool, along with the assigned weights and priority levels, reflect stakeholder preferences and support the overarching intent of our MCDA process, to assess and prioritize based on the importance and feasibility of RWE proposals [12]. Third, through the iterative development of the MCDA process, we have identified multidisciplinary roles of key stakeholders involved in cancer drug funding to aid in the application and implementation of the tool [12,16]. Combined, these differences align with the general consensus around the need for developing MCDA fit-for-purpose to support the decision context and are reflective of stakeholder preferences [12,15,16].

Several strengths and practical considerations emerged during our validation exercise. First and foremost, we established guidelines for the application of the MCDA rating tool and an approach to support the validation exercise, which was critical to the realization of a structured deliberative process incorporating relevant perspectives in the application of MCDA to inform HTA reassessment. Our guideline provides guidance on issues such as committee composition (e.g., expertise and roles required), format and processes (e.g., consensus building and duration of meetings), evidence sources (e.g., information derived from credible sources), and data collection and reporting practices (e.g., documenting consensus scores to aid in transparent decision-making), aligning with the best practices for deliberative processes published by ISPOR following our validation exercise [17]. Understanding the current landscape, we developed an MCDA process that is reflective of the values and preferences of decision-makers and encompasses resources that are readily available in an effort to reduce cognitive and resource burden and to ease implementation. Second, the importance of an iterative process in developing an MCDA rating tool and ensuring that criteria included within the rating tool are reflective of the values of relevant stakeholders and applicable to the decision context cannot be stressed enough. Although the MCDA rating tool was validated previously through two pilot tests, our validation exercise identified several additional opportunities for refinement. A wider stakeholder group helped to operationalize the rating tool, allowing for consistent interpretation and transparency in the decision-making process. Third, one of the strengths of our MCDA process is the versatility of our MCDA rating tool, which can be leveraged throughout the life-cycle evaluation of cancer therapies. For instance, our MCDA rating tool can help to inform whether a time-limited recommendation conditional on RWE generation is appropriate by determining whether an RWE analysis can be feasibly conducted in a timely manner. Further, our MCDA rating tool can help to inform post-market drug evaluation in identifying and prioritizing relevant uncertainties that are of importance to decision-makers, which can be feasibly addressed through RWE evaluation. The refinements and lessons learned throughout our validation exercise will help inform next steps as the PDS-WG focuses on exploring opportunities to help support the integration of RWE to inform policy decisions. We believe the current MCDA rating tool could be considered for adoption, provided context-specific adaptions, to help prioritize important and feasible RWE studies in determining whether a drug used in the treatment of cancer in regular clinical practice shows a similar benefit to what was demonstrated in prior clinical trials. Jurisdictions should discuss how RWE can be applied in order to align future policies that incorporate RWE. In addition, it could be applied to determine if drugs should remain on formulary based on the findings of the RWE analysis.

## 7. Conclusions

Our collaboration with PAG in evaluating the application of MCDA for research prioritization proved to be a valuable experience. Direct feedback from potential users of the prioritization process helped to improve the utility of the rating tool and deliberative processes. Lessons learned from this experience coupled with the need for a structured deliberative process involving a diverse stakeholder group will help guide HTA agencies, such as CADTH, in developing formalized processes for research prioritization both in Canada and internationally.

## Figures and Tables

**Table 1:** RWE proposals assessed during the MCDA validation exercise.

Validation Exercise Meeting Dates	RWE Proposal	Research Question(s)
18 August 2021	Polatuzumab vedotin for relapsed/refractory diffuse large B-cell lymphoma	What is the real-world comparative effectiveness **(overall survival)** of polatuzumab vedotin in combination with bendamustine and rituximab in patients with relapsed/refractory diffuse large B-cell lymphoma (DLBCL) who are not eligible for autologous stem cell transplantation (ASCT) and have received at least one prior therapy, as compared to standard systemic therapy?
22 November 2021	Dabrafenib and Trametinib for BRAF V600E mutation Positive Metastatic Non-Small Cell Lung Cancer	What is the real-world **progression-free survival** (RQ1) of dabrafenib and trametinib as first line-line systemic therapy in patients with BRAF V600E mutation positive metastatic non-small cell lung cancer (NSCLC), as compared to standard platinum-doublet chemotherapy +/− pembrolizumab?
		What is the real-world **overall survival** (RQ2) of dabrafenib and trametinib as first line-line systemic therapy in patients with BRAF V600E mutation positive metastatic non-small cell lung cancer (NSCLC), as compared to standard platinum-doublet chemotherapy +/− pembrolizumab?
		What is the real-world **time to next treatment** (RQ3) of dabrafenib and trametinib as first line-line systemic therapy in patients with BRAF V600E mutation positive metastatic non-small cell lung cancer (NSCLC), as compared to standard platinum-doublet chemotherapy +/− pembrolizumab?
		What is the real-world **duration of treatment** (RQ4) of dabrafenib and trametinib as first line-line systemic therapy in patients with BRAF V600E mutation positive metastatic non-small cell lung cancer (NSCLC), as compared to standard platinum-doublet chemotherapy +/− pembrolizumab?
23 February 2022	Nivolumab in relapsed/ Refractory Classical Hodgkin’s lymphoma	What is the real-world **overall survival** of nivolumab in patients with classical Hodgkin’s lymphoma with evidence of disease progression following autologous stem cell transplantation (ASCT) and brentuximab vedotin (BV), as compared to standard single-agent chemotherapy or pembrolizumab immunotherapy? *
	Atezolizumab in combination with bevacizumab for unresectable/metastatic hepatocellular carcinoma	What is the real-world **overall survival** of atezolizumab in combination with bevacizumab as first-line treatment for patients with unresectable or metastatic hepatocellular carcinoma (HCC), as compared to either sorafenib or lenvatinib?
23 June 2022	Durvalumab in Combination with Platinum Etoposide for Extensive-Stage Small Cell Lung Cancer	What is the real-world **overall survival** of durvalumab in combination with platinum-etoposide as first-line treatment for patients with extensive-stage small cell lung cancer, as compared to platinum-etoposide alone?
	Nivolumab in Combination with Fluoropyrimidine and Platinum-containing Chemotherapy for Metastatic Gastric Adenocarcinoma	What is the real-world **overall survival** of nivolumab in combination with fluoropyrimidine and platinum-containing chemotherapy as first-line systemic therapy for HER-2 negative locally advanced or metastatic gastric adenocarcinoma, as compared to fluoropyrimidine and platinum-containing chemotherapy alone?
5 December 2022	Nivolumab in Combination with Fluoropyrimidine and Platinum-containing Chemotherapy for Metastatic Gastric Adenocarcinoma ^†^	What is the real-world **overall survival** of nivolumab in combination with fluoropyrimidine and platinum-containing chemotherapy as first-line systemic therapy for HER-2 negative locally advanced or metastatic gastric adenocarcinoma, as compared to fluoropyrimidine and platinum-containing chemotherapy alone?

Note: Endpoint(s) of interest are bolded. * RWE Proposal, including the research question, was evaluated previously in an MCDA pilot study reported by Parmar et al., 2023 [13]. ^†^ Previously reviewed during the June 2022 meeting.

**Table 2:** MCDA validation exercise outcomes.

Meeting Dates	RWE Proposal	Endpoint	Consensus Score *			
			Importance	Feasibility	Total	Priority
18 August 2021	Polatuzumab vedotin for relapsed/refractory diffuse large B-cell lymphoma	OS	122	104	226	High
22 November 2021	Dabrafenib and trametinib for BRAF V600E mutation Positive Metastatic Non-Small Cell Lung Cancer	PFS	87	98	185	Low
		OS	124	133	256	High
		TTNT	68	129	198	Low
		DOT	n/a ^†^	n/a ^†^	n/a ^†^	n/a ^†^
23 February 2022	Nivolumab in relapsed/refractory classical Hodgkin’s lymphoma	OS	105	115	220	Medium
	Atezolizumab in combination with Bevacizumab for unresectable/metastatic hepatocellular carcinoma	OS	105	107	212	Medium
23 June 2022	Durvalumab in Combination with Platinum Etoposide for Extensive-Stage Small Cell Lung Cancer	OS	68	141	209	Medium
	Nivolumab in Combination with Fluoropyrimidine and Platinum-containing Chemotherapy for Metastatic Gastric Adenocarcinoma	OS	58	127	185	Low
5 December 2022	Nivolumab in Combination with Fluoropyrimidine and Platinum-containing Chemotherapy for Metastatic Gastric Adenocarcinoma ^‡^	OS	77	127	204	Low

Abbreviations: OS, Overall survival; PFS, Progression free survival; TTNT, Time to next treatment; DOT, Duration of treatment; n/a, not applicable. * The consensus score represents an aggregated weighted score for an individual RWE proposal ranging from 100 to 300, with a larger score indicating greater value and feasibility [Parmar et al., 2023] [13]. ^†^ Due to time constraints, research questions were not assessed. ^‡^ Previously reviewed in the June 2022 meeting.

## Data Availability

Data is contained within the article.

## Appendix A. Initial Set of MCDA Criteria

**Table A1:** Initial MCDA Rating Tool (taken from Parmar et al., 2023) [13].

Criteria	Rating Scale			Weight
	1	2	3	
**Group A—Criteria to Assess the Importance of the RWE Question**				
**Drug’s perceived incremental benefit:** The objective of this criterion is to evaluate the perceived clinical benefit of the therapy compared to existing options. The ‘perceived’ clinical benefit is based on the currently available objective evidence (including primary clinical trial data and indirect comparisons ^1^) and expected long-term outcomes (in the setting of immature data).	Rated using the European Society of Medical Oncology (ESMO) Magnitude of Clinical Benefit Scale (MCBS) v1.0. [17]			17.65
	**Minimal to low clinically meaningful incremental benefit,** as evidenced by: (a) overall survival benefit demonstrated through a hazard ratio >0.65 and/or gain in median overall survival of <2.5 months, as compared to current standard(s) of care, for therapies with a median overall survival of <1 year; or (b) overall survival benefit demonstrated through a hazard ratio of >0.70 and/or gain in median overall survival of <3 months, as compared to current standard(s) of care, for therapies with a median overall survival > 1 year; or (c) median progression free survival benefit demonstrated through a hazard ratio of >0.65, as compared to current standard(s) of care; or) clinical benefit demonstrated in an alternative outcome (including improvements in response rate, quality-of-life or other clinically meaningful outcome).	**Moderate clinically meaningful incremental benefit,** as evidenced by: (a) survival benefit demonstrated by a hazard ratio ≤0.65 and gain in median survival of 2.5–2.9 months, as compared to current standard(s) of care, for therapies with median overall survival ≤ 1 year; or (b) overall survival benefit demonstrated by a hazard ratio ≤0.70 and gain in median overall survival of 3–4.9 months, as compared to current standard(s) of care, for therapies with median overall survival > 1 year; or (c) progression-free survival benefit demonstrated by a hazard ratio ≤0.65 and gain ≥1.5 months, as compared to current standard(s) of care.	**Substantial clinically meaningful incremental benefit,** as evidenced by: (a) overall survival benefit demonstrated through a hazard ratio of <0.65 and gain in median overall survival of 2.5–3.0 months, as compared to current standard(s) of care, for therapies with median overall survival < 1 year; or (b) overall survival benefit demonstrated through a hazard ratio ≤ 0.70 and gain in median overall survival of ≥5 months, as compared to current standard(s) of care, for therapies with median overall survival > 1 year.	
**Magnitude of uncertainty:** The objective of this criterion is to assess the degree of uncertainty in question (the uncertainty can be about toxicity, clinical effectiveness, quality-of-life, treatment sequence, generalizability of benefits, costs or other).	**Minimal uncertainty:** This can be based upon either a qualitative assessment or quantitative assessment (the latter can be conceptualized as a <10% variation in either of the following: (a) the confidence intervals around the survival estimates; (b) the upper and lower range of ICERs from the pCODR assessment ^1^).	**Moderate uncertainty:** This can be based upon either a qualitative assessment or quantitative assessment (the latter can be conceptualized as a 10–25% variation in either of the following: (a) the confidence intervals around the survival estimates; (b) the upper and lower range of ICERs from the pCODR assessment ^1^).	**Substantial uncertainty:** This can be based upon either a qualitative assessment or quantitative assessment (the latter can be conceptualized as a >25% variation in either of the following: (a) the confidence intervals around the survival estimates; (b) the upper and lower range of ICERs from the pCODR assessment ^1^).	10.6
**Relevance of uncertainty:** The objective of this criterion is to assess the relevance of resolving the uncertainty to decision-makers (i.e., what is the likelihood that resolving the uncertainty with new evidence will alter the funding status or clinical treatment recommendations).	**Indirect relevance:** As assessed by expert opinions, there is an expected low likelihood for new evidence to facilitate a change in funding status (i.e., facilitate drug price re-negotiations) and/or change in clinical treatment recommendations (i.e., indicated patient populations or treatment sequence).	**Moderate relevance:** As assessed by expert opinions, there is uncertainty in the likelihood for new evidence to facilitate a change in funding status (i.e., facilitate drug price re-negotiations) and/or change in clinical treatment recommendations (i.e., indicated patient populations or treatment sequence).	**Substantial relevance:** As assessed by expert opinions, there is an expected high likelihood for new evidence to facilitate a change in funding status (i.e., facilitate drug price re-negotiations) and/or change in clinical treatment recommendations (i.e., indicated patient populations or treatment sequence).	18.8
**Group B—Criteria to Assess the Feasibility of the RWE Project**				
**Comparator:** The objective of this criterion is to assess the likelihood that a relevant historical or contemporaneous Canadian comparator population, of sufficient size, can be identified within a reasonable time frame (i.e., within time to be relevant to the funding decision, for contemporaneous control). A ‘relevant’ comparator is a group that has been treated according to current Canadian standard of care regimen.	**Substantial concern:** Unlikely to identify an appropriate comparator population within a reasonable time due to absence of clear standard-of-care therapy (i.e., >2 relevant standard-of-care treatments currently available or evolving standard-of-care treatment) and/or low-volume patient population.	**Moderate concern:** Moderate concern for the identification of an appropriate comparator population due to absence of clear standard-of-care therapy (i.e., 2 relevant standard-of-care treatments currently available) and/or moderate-volume patient population.	**Low concern:** Appropriate comparator population will be easily identified due to a well-defined standard of care therapy and high-volume patient population.	11.8
**Cases:** The objective of this criterion is to assess the likelihood that there will be enough patients receiving the treatment in question to have a sufficient sample size within a reasonable time frame (i.e., within time to be relevant to the funding decision).	**Substantial concern:** Unlikely to establish a sufficient sample size (with appropriate follow-up for relevant outcome(s)) within a reasonable time ^2^ based upon expected incidence of disease (using Canadian provincial estimates) and required sample size for analysis.	**Moderate concern:** Likely to establish a sufficient sample size, based upon expected incidence of disease (using Canadian provincial estimates) but unlikely to have follow-up for relevant outcome(s) within a reasonable time ^2^ based upon expected incidence (using Canadian provincial estimates) and required sample size for analysis.	**Low concern:** Very likely to establish a sufficient sample size (with appropriate follow-up for relevant outcome(s)) within a reasonable time ^2^ based upon expected incidence of disease (using Canadian provincial estimates) and required sample size for analysis.	14.1
**Data:** The objective of this criterion is to assess the quality of data available in at least one Canadian province to address the uncertainty. This requires an assessment of the availability and completeness of data for both the exposed and comparator cohorts pertaining to: (a) data for relevant patient and disease characteristics to account for important co-variates, ensure un-biased comparability between groups and measure relevant outcomes +/− (b) data for relevant costing inclusive of total health care costs accrued during treatment (ex. systemic treatment, planned and unplanned health care resource utilization).	**Substantial concern:** Substantial concern for the availability of high-quality and complete data for both exposed and comparator cohorts in known real-world databases (as assessed by an absence of ≥1 of the following: (a) patient and/or disease characteristics required to define current funding eligibility; (b) >2 relevant patient and/or disease co-variates; (c) ability to identify primary systemic treatment, inclusive of line-of-therapy).	**Moderate concern:** Moderate concern for the availability of high-quality and complete data for both exposed and comparator cohorts in known real-world databases (as assessed by an absence of ≥1 of the following: (a) 1–2 relevant patient and/or disease co-variates; (b) ability to identify prior or subsequent treatment inclusive of line-of-therapy).	**Low concern:** No expected issues in accessing high-quality and complete data in known real-world databases.	17.65
**Expertise and Methodology:** The objective of this criterion is to evaluate the availability of required expertise (ex. clinical experts, data analysts and methodologists) and methodology to conduct the study.	**Substantial concern:** Expected challenges to find the necessary expertise and need to develop new methods to conduct the study, with above limitations in data taken into consideration (if applicable).	**Moderate concern:** Expected challenges to find the necessary expertise or need to develop new methods to conduct the study, with above limitation in data taken into consideration (if applicable).	**Low concern:** No expected issues with the availability of the necessary expertise and no new methods required to conduct the study.	9.4

^1^ Please refer to the pCODR economic guidance report for details on pCODR’s reanalyzed ICERs. ^2^ Reasonable time: time required is less than half the time of remaining patency duration. Abbreviations: ICER: incremental cost-effectiveness ratio; pCODR: pan-Canadian Oncology Drug Review.
